# Time from blood draw to multiple electrode aggregometry and association with platelet reactivity

**DOI:** 10.1007/s11239-022-02720-7

**Published:** 2022-11-08

**Authors:** David Hesselbarth, Diona Gjermeni, Sofia Szabo, Patrick M. Siegel, Philipp Diehl, Martin Moser, Christoph Bode, Christoph B. Olivier

**Affiliations:** 1 Department of Cardiology and Angiology, University Heart Center Freiburg-Bad Krozingen, Faculty of Medicine, University of Freiburg, Hugstetter Str. 55, Freiburg, 79106 Germany; 2 Department of Cardiology, Pneumology, Angiology, Geriatrics & Intensive Care Medicine, Ortenau Hospital, Lahr-Ettenheim, Germany

**Keywords:** Antithrombotic therapy, Coronary syndrome, Platelet reactivity, Multiple electrode aggregometry

## Abstract

Results from multiple electrode aggregometry (MEA) may vary according to pre-analytic factors. This study aimed to analyze the association of time from blood draw to MEA in patients undergoing percutaneous coronary intervention (PCI). In this observational single-center cohort study, platelet aggregation (aggregation units, U) was quantified by MEA (Multiplate Analyzer) after stimulation with adenosine diphosphate (ADP; final concentration [Fc] 6.4 μM), thrombin receptor activating peptide (TRAP; Fc 32 μM), or arachidonic acid (AA; Fc 0.5 mM) in patients treated with ASA and clopidogrel following PCI. High on-clopidogrel platelet reactivity (HPR) was defined as ADP-induced platelet aggregation ≥ 46 U. The manufacturer recommends performing the analysis within 30–180 min after blood draw. Patients were grouped according to the time from blood draw to MEA: 30–180 min, < 30 min, or > 180 min. Platelet function of 273 patients with coronary artery disease undergoing PCI with dual antiplatelet therapy was analyzed. The median age was 72 years (interquartile range, IQR 62–79) and 179 (66%) were male. Median ADP-, TRAP-, and AA-induced aggregation was 25 (IQR 18–36) U, 79 (IQR 63–96) U, and 12 (IQR 7–18) U, respectively. For those analyzed within 30–180 min from blood draw, no significant correlation of time from blood draw to MEA was observed 1) ADP (r = − 0.04, p = 0.51); 2) TRAP (r = − 0.06, p = 0.32); 3) AA (r = − 0.03, p = 0.67). In patients undergoing percutaneous coronary intervention and treated with dual antiplatelet therapy, the time from blood draw to multiple electrode aggregometry does not correlate with ADP- induced aggregation when the measurement occurred within the recommended time interval of 30–180 min after blood draw.

**Supplementary Information:**

The online version contains supplementary material available at 10.1007/s11239-022-02720-7.

## Highlights

- This study aimed to analyze the association of time from blood draw to multiple electrode aggregometry in patients with coronary artery disease. The manufacturer recommends performing the analysis within 30–180 min after blood draw.
- In 273 patients with dual antiplatlet therapy after percutaneous coronary intervention, the time from blood draw to multiple electrode aggregometry did not correlate with ADP- induced aggregation when the measurement occurred within the recommended time interval of 30–180 min after blood draw.
- Multiple electrode aggregometry produces consistent results of ADP-induced platelet aggregation independent of the time from blood draw to measurement within 30–180 min.
- This study suggests that time from blood draw to multiple electrode aggregometry does not impact platelet aggregation results for potential clinical decision making in patients receiving clopidogrel.

## Background

Guidelines recommend dual antiplatelet therapy (DAPT) with acetylsalicylic acid (ASA) and a P2Y_12_ inhibitor for most patients after percutaneous coronary intervention (PCI). Compared with clopidogrel, prasugrel and ticagrelor more strongly inhibit platelet function [1] and prevent ischemic events in patients after acute coronary syndrome [2] at the cost of an increased risk of bleeding [3]. High risk of recurrent ischemic events in patients treated with clopidogrel is partially determined by the inter-individual variability of its activity [4]. Platelet function tests might help tailor DAPT therapy.

Guidelines or consensus documents suggest multiple electrode aggregometry (MEA) to guide platelet inhibition in specific clinical scenarios especially for patients with high thrombotic or bleeding risk and acute coronary syndrome when DAPT therapy for 12 months is not sustainable [5–7].

To identify patients with high on-clopidogrel platelet reactivity (HPR) specific cut-off values have been determined such as an ADP-induced aggregation ≥ 46 U when assessed by MEA [6]. HPR presents in up to 30% of patients undergoing PCI and associates with higher risk for ischemic events [6].^.^ The expert consensus and guidelines on platelet function testing also suggest the use of MEA as a guidance tool to escalate antiplatelet therapy in specific groups of patients with high ischemic risk [6, 7].

However, results of MEA vary according to patient characteristics and may also depend on pre-analytic factors [8]. Previous studies with small sample sizes and predominant healthy patients performed MEA analysis at different time points since blood draw and showed conflicting results of platelet aggregation [9, 10]. Data from a larger cohort of patients with comorbidities and medication in clinical practice is needed.

Blood storage can deteriorate platelet function over time [11], thus we hypothesize that platelet function alters after blood draw and MEA would reflect these changes over time. Our study aimed to analyze the association of time from blood draw to MEA in patients undergoing PCI and receiving DAPT.

## Methods

### Study design and clinical characteristics

We analyzed data from an observational single-center cohort study performed at Heart Center Freiburg University [1, 11]. The study was performed in accordance with the Helsinki declaration and was approved by the ethics committee of the Albert-Ludwigs-University Freiburg, Germany (Registry Number 183/07). All patients had given their written informed consent. 359 patients who underwent coronary stent implantation and were treated with 100 mg ASA and 75 mg clopidogrel per standard-of-care were included prospectively. Patients with coagulation disorders such as antiphospholipid syndrome were not included in the study. Medical history, laboratory values and medication were extracted from the clinical charts. Peri-procedural medication or coronary intervention characteristics were collected from the intervention protocols. All patients received a loading with clopidogrel of 300 mg (at least 24 h before platelet aggregation assay) or 600 mg (at least 12 h before platelet aggregation assay). All patients received dual antiplatelet maintenance therapy with 100 mg ASA and 75 mg clopidogrel per day. When there was no indication to continuous oral anticoagulation, ASA was given permanently after stent implantation.

For the present analysis, we excluded patients in whom data such as time of blood draw, time of measurement or both as well as information regarding antiplatelet therapy were missing. We analyzed a subgroup of 273 patients presenting with coronary artery disease and under DAPT. The manufacturer recommends performing the analysis within 30–180 min after blood draw [12].Patients were grouped according to the time from blood draw to MEA: (1) 30–180 min (2) < 30 min (3) > 180 min.

### Blood samples

Venous blood was collected using a 21 G butterfly needle (Safety-Multifly®-Set, Sarstedt, Nümbrecht, Germany) to a Fc of > 15 μg/ml r-hirudin (SARSTEDT Monovetten, Nümbrecht, Germany). When possible, blood samples were analyzed within the first three hours after blood draw in order to prevent platelet aggregation caused by storage. Measurements were performed within 30 min after venous puncture when aggregometry measurement was indicated by the physician. Blood samples were stored at room temperature until the analysis.

### Catheter procedure and hospitalization

Patients with ST-elevation myocardial infarction received coronary angiography and PCI as soon as their arrival to the hospital. Patients with non-ST-elevation myocardial infarction (positive troponin) or unstable angina pectoris received coronary angiography within 48 h from presenting in the clinic. Arterial access was chosen at the discretion of the interventionalist. Oral anticoagulation was withheld and recommenced 12 h after the procedure. All patients received heparin for anticoagulation per standard of care. All patients received at least one drug-eluting stent (DES) implantation. Pre- and post-dilatation were performed according to the judgment of the interventionalist. A successful procedure was considered TIMI flow > 2 post-intervention.

### Platelet aggregometry

Multiple electrode aggregometry (MEA, Roche Diagnostics, Switzerland) was performed with whole hirudin anticoagulated blood solution on the day of PCI or the day after.

MEA is a whole blood, platelet function assay detecting changes in electrical impedance when platelets aggregate on electrodes. Changes in electric impedance are transformed into arbitrary aggregation units (U). Three different activators (adenosine diphosphate (ADP), arachidonic acid adenosine (AA), and thrombin receptor activating peptide-6 (TRAP-6) are used to activate the patient’s platelets. ADP- and AA are used to monitor therapy with clopidogrel and aspirin (ASA). To assess the overall platelet aggregability, blood samples were stimulated with TRAP-6 (Fc 32 μM). To specifically quantify the effect of P2Y_12_-inhibitors and aspirin, whole blood was stimulated with ADP (Fc 6.4 μM) and arachidonic acid (Fc 0.5 mM).

Clinical studies have demonstrated that HPR in patients receiving clopidogrel is associated with recurrent cardiovascular events [13]. HPR under clopidogrel as assessed by MEA was defined as ADP-induced aggregation ≥ 46 U. Stimulation with thrombin receptor activating peptide-6 (TRAP) served as a positive control [14]. ADP- was normalized to TRAP-induced platelet aggregation (relative-ADP-induced aggregation; r-ADP) [15].

### Statistical analysis

Data are presented as numbers with frequencies for categorical variables and medians with interquartile range (IQR) for continuous variables. Kruskal–Wallis test was used to compare the median distribution for continuous variables. To assess if there was an association between the time from blood draw until MEA and MEA results a correlation test was performed. Pearson’s correlation coefficients were used for bivariate correlational analyses. To assess the association of demographic and procedural variables with MEA we performed a multiple linear regression. All tests were 2-tailed and p values ≤ 0.05 were considered statistically significant. Data were analyzed with Prism 9.2.0 (GraphPad Software, La Jolla, California, USA) and SPSS 27.0.0.1 (SPSS Inc, Chicago, Illinois, USA).

## Results

273 measurements from 273 patients with coronary artery disease undergoing PCI with DAPT were analyzed. The median age of the patients was 72 years (IQR 62–79) and 179 (66%) were male. 69 (25%) had a history of myocardial infarction and 31 (11%) had a history of stroke. 56% of the patients presented with a known reduced left ventricular function. 219 (80%) patients had arterial hypertension and 175 (64%) had hyperlipidemia.

MEA was performed for 176 (64%) of the patients either on the day of PCI or the day after. Median (IQR) time to MEA was 65 (45–113) min and ranged from 10 to 580 min. 245 measurements (90%) were performed within 30–180 min as recommended by the manufacturer of the aggregometer, 5 (2%) measurements in less than 30 min after blood draw, and 23 (8%) measurements in more than 180 min after blood draw. Overall, median ADP-, TRAP-, AA-induced aggregation and r-ADP were 25 (IQR 18–36) U, 79 (IQR 63–96) U, 12 (IQR 7–18) U and 35 (IQR 25–44) %, respectively.

**Fig. 1:**
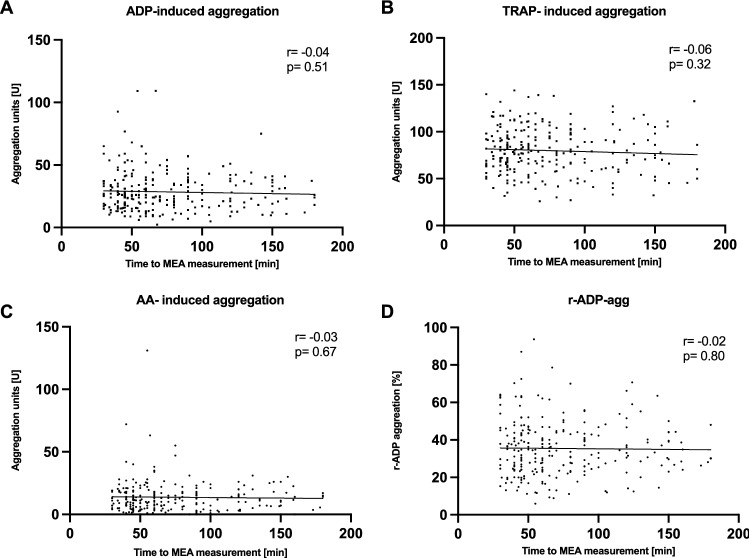
Multiple electrode aggregometry performed within the manufacturers’ recommendations (30–180 min) demonstrated no relevant correlation from time to blood draw to MEA with ADP-, TRAP-, AA-induced aggregation and r-ADP aggregation (**A**–**D**)

HPR was observed in 34 patients (12%). For those analyzed within 30–180 min from blood draw, no relevant correlation of time from blood draw to MEA was observed (Fig. 1) (A) ADP (r = − 0.04, p = 0.51); (B) TRAP (r = − 0.06, p = 0.32); (C) AA (r = − 0.03, p = 0.67); (D) r-ADP (r = − 0.02, p = 0.80).

**Fig. 2:**
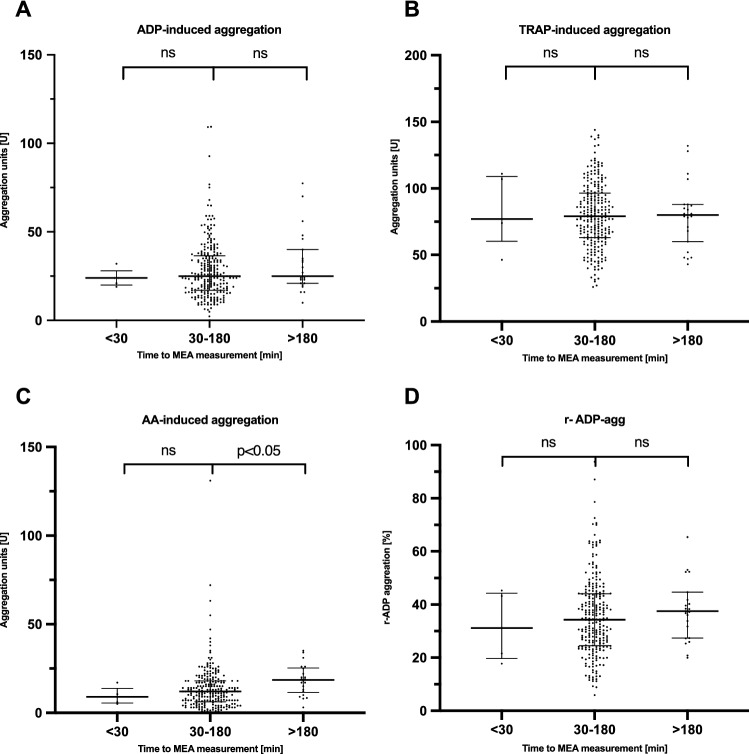
Median ADP- TRAP-, induced aggregation and median r-ADP aggregation (**A**, **B**, **D**) were not significantly different when performed outside the manufacturers’ recommended timeframe (30–180 min) to MEA measurement. AA-induced aggregation when measured > 180 min was significantly increased (**C**)

Median ADP-, TRAP-, AA-induced aggregation, and r-ADP performed within the manufacturers´ recommendations were 25 (IQR 17–36) U; 79 (IQR 63–96) U; 12 (IQR 6–18) U and 34 (IQR 25–44) U, respectively. TRAP-, ADP- induced and r-ADP measurements performed outside these recommendations (< 30 min or > 180 min) were not significantly different from those performed within (Fig. 2).

(A) ADP < 30 min: 24 (IQR 20–28) U, p = 0.40, > 180 min: 25 (IQR 21–40) U p = 0.91; (B) TRAP < 30 min: 77 (IQR 60–109) U, p = 1.0, > 180 min: 80 (IQR 60–88) U, p = 0.97; (D) r-ADP < 30 min: 31 (IQR 20–44) %, p = 1.0, > 180 min: 37 (IQR 27–45) %, p = 0.19.

Compared with AA-induced aggregation within the recommended time (12 [IQR 6–18])U, aggregation measured > 180 min after the blood draw was increased (AA > 180 min: 18 [IQR 11–25]) U, p = 0.04 (Fig. 1, C). This difference was not consistent when analyzed with multiple linear regression (p = 0.17; supplemental Table S1).

## Discussion

The main finding is that time from blood draw until MEA did not correlate with MEA results in patients receiving DAPT with ASA and clopidogrel. Neither when the measurement occurred within the recommended time interval of 30–180 min after blood draw, nor when it occurred outside of this interval.

MEA is a standardized method and a tool to guide individual therapy in selected scenarios [6]. Compared to light transmission aggregometry (LTA), the historical gold standard, MEA is a more standardized assay with a fixed dose of agonists.

However, preanalytical variables influence the extent of platelet inhibition and may confound the results. Platelets can be activated during the process of the blood draw or their function is altered during storage [16]. Other studies identified the body mass index and platelet count as relevant factors for platelet reactivity [8, 17]. A retrospective analysis of 359 patients after coronary stent implantation treated with aspirin and clopidogrel demonstrated a significant correlation of TRAP- and ADP- induced aggregation with platelet count and white blood cell count, while this correlation was not found for r-ADP [8]. Additionally, the choice of anticoagulant in the sample tubes influences the measurement of platelet reactivity [10].

Recent studies analyzed the correlation of ADP- induced platelet aggregation with time to MEA measurement with conflicting results. In 11 patients, treated with clopidogrel there was a relevant time-dependent decrease of ADP–induced platelet reactivity (120 min vs. 30 min: − 34% and 240 min vs. 30 min: − 49%) [9]. In contrast, healthy volunteers had a significant increase in ADP- platelet reactivity when the time since blood collection passed, however with a minor clinical relevance of 10% increase (180 min vs 30 min) [10].

In healthy volunteers, TRAP-induced platelet reactivity was significantly but clinically acceptable reduced (− 14%; 180 min vs. 30 min) [10]. Another analysis of 21 samples showed a time-dependent decrease of ADP-, TRAP-, and AA-induced-aggregation, however with acceptable stability between 30 and 120 min [11].

In general, platelet reactivity, when measured within 30–180 min after blood collection, seems stable. Most studies observed a reduction in platelet reactivity within the first 30 min after blood draw, which can be a consequence of platelet aggregation within this period [11]. The reactivity peaked at 30 min and was followed by a time-dependent decrease of platelet reactivity, however within a clinically acceptable range [10, 11, 14]. Hirudin is the recommended anticoagulant in sample tubes for MEA. However, compared to citrate or EDTA, platelet aggregation is more pronounced when hirudin is used and may contribute to a time-dependent reduction observed by some authors [10, 11]. Incomplete platelet inhibition in the sample tubes may lead to platelet aggregation before MEA measurement which may result in time-dependent reduced platelet reactivity [11].

In our cohort of 273 patients with coronary artery disease receiving DAPT we observed no correlation of time from blood draw with platelet aggregation after activation with ADP, TRAP, and AA within 30–180 min (Fig. 1A–D). This finding is consistent with other studies, reporting only minor and clinically not relevant changes of time-dependent platelet reactivity within this interval.

We also found no difference of ADP- and TRAP-induced aggregation and r-ADP when the measurement was performed outside the recommended time interval (< 30 min or > 180 min; Fig. 2A–D), which is inconsistent with previously performed studies. However, most of these studies were conducted with small sample sizes and predominant healthy subjects not taking clopidogrel, ASA, or other medication. Effects of platelet hyperreactivity or platelet dysfunction may be attenuated by platelet inhibitors. Similarly, time from blood draw to MEA was not independently explanatory for the variance in AA-induced platelet aggregation. Due to the small number of measurements performed outside the recommended windows a type II error cannot be excluded [18]. Table 1 indicates differences among subgroups that potential confound results.

**Table 1:** Subgroup specific clinical baseline characteristics

Baseline characteristics	Total		30–180 min		< 30 min		> 180 min		p-value
	n = 273		n = 245		n = 5		n = 23		
*Demographics*									
Male sex	179	66%	160	65%	4	80%	15	65%	
Age (years)	72	[62–79]	72	[62–80]	70	[66–76]	74	[70–78]	
*Medical history*									
Previous MI	69	25%	61	25%	2	40%	6	26%	0.74
Reduced LV-function	153	56%	133	54%	4	80%	16	70%	0.13
Atrial fibrillation	52	19%	47	19%	2	40%	3	13%	0.11
Stroke/TIA	31	11%	26	11%	1	20%	4	17%	0.51
GI-Bleeding	8	3%	7	3%	0	0%	1	4%	0.85
PAD	34	13%	31	13%	0	0%	3	13%	0.69
COPD	15	6%	13	5%	1	20%	1	4%	0.35
*Cardiovascular risk factors*									
Hyperlipidemia	175	64%	153	62%	4	80%	18	78%	0.24
Aterial hypertension	219	80%	194	79%	4	80%	21	91%	0.38
Diabetes mellitus	78	29%	72	29%	1	20%	5	22%	0.88
Family history	59	22%	52	21%	2	40%	5	22%	0.60
History of smoking	114	42%	105	43%	1	20%	8	35%	0.01
*Procedural characteristics*									
Index event of PCI									0.02
Elective	156	57%	147	60%	2	40%	7	30%	
Acute	117	43%	98	40%	3	60%	16	70%	

The values are n (%) or median (interquartile range)

*MI* myocardial infarction, *LV-function* left ventricular-function, *TIA* transient ischemic attack, *GI-Bleeding* gastrointestinal bleeding, *PAD* peripheral artery disease, *COPD* chronic obstructive pulmonary disease, *PCI* percutaneous coronary intervention

Current guidelines [2, 7] and expert consensus documents [6] suggest the use of platelet functioning tests such as MEA in patients with high ischemic or bleeding risk to tailor antiplatelet therapy in patients undergoing PCI. This study provides real-world data of patients receiving platelet inhibitors. These insights may contribute to clinical decision-making of MEA-guided platelet therapy in clinical scenarios such as urgent PCI during night shift when timely measurement is not possible.

### Limitations of the study

We analyzed different patients with different time intervals from blood draw to measurement, therefore potentially individual patient characteristics as a confounding factor cannot be excluded. Compared to previously performed studies with sample sizes of 10–20 predominantly healthy individuals, we analyzed a large cohort of 273 patients with clinically relevant comorbidities and DAPT. However, the measurement performed outside the recommended time interval was performed with a small sample size (< 30 min: n = 5; > 180: n = 23). Thus, evidence of these measurements may be limited and should be examined in a larger patient cohort.

## Conclusion

Multiple electrode aggregometry produces consistent results of ADP-induced platelet aggregation independent of the time from blood draw to measurement within 30–180 min. Although other studies observed decreasing platelet aggregation over time in healthy volunteers, this study suggests that time from blood draw to multiple electrode aggregometry does not impact platelet aggregation results for potential clinical decision making in patients receiving clopidogrel.

## Supplementary Information

Below is the link to the electronic supplementary material.

Supplementary file1 (DOCX 29 kb)
