# Supplementary material for: Time from blood draw to multiple electrode aggregometry and association with platelet reactivity
Source: J Thromb Thrombolysis. 2022 Nov 8;55(1):134–40. doi: 10.1007/s11239-022-02720-7 (PMC9925483; doi:10.1007/s11239-022-02720-7)
Supplement: Supplementary file 1 — Supplementary file1 (DOCX 29 kb) [file 11239_2022_2720_MOESM1_ESM.docx]

**Supplementary Information**

| **Characteristics**  **(n=273)** | | **Regression-**  **coefficient** | **Std.-**  **deviation** | **Beta**  **coefficient** | **95% CI** | **p-value** |
| --- | --- | --- | --- | --- | --- | --- |
| **Demographics** | |  |  |  |  |  |
|  | Male sex | -6.49 | 16.80 | -0.03 | -39.60;26.60 | 0.70 |
|  | Age [years] | 0.49 | 0.76 | 0.05 | -1.03;2.00 | 0.53 |
| **Medical history** | |  |  |  |  |  |
|  | Previous MI | -31.61 | 18.66 | -0.1 | -62.39;127.99 | 0.09 |
|  | Reduced LV-function | 3.83 | 8.07 | 0.03 | -12.05;19.73 | 0.64 |
|  | Atrial fibrillation | -9.51 | 10.18 | -0.06 | -29.56;10.53 | 0.35 |
|  | Stroke/TIA | 64.32 | 25.90 | 0.16 | 13.31;115.34 | 0.01 |
|  | GI-Bleeding | 32.79 | 48.33 | 0.05 | -62.39;127.99 | 0.50 |
|  | PAD | -12.56 | 23.58 | -0.03 | -59.00;33,88 | 0.60 |
| **Cardiovascular risk factors** | |  |  |  |  |  |
|  | Hyperlipidemia | -1.56 | 16.935 | -0.01 | -34.91;31.80 | 0.93 |
|  | Aterial hypertension | -30.61 | 20.55 | -0.09 | -71.09;9.87 | 0.14 |
|  | Diabetes mellitus | 10.16 | 9.75 | 0.07 | -9,04;29,37 | 0.30 |
|  | Family history | -19.81 | 19.30 | -0.07 | -57.83;18.20 | 0.30 |
|  | History of smoking | 18.67 | 16.15 | 0.08 | -13.13;50.47 | 0.245 |
| **Procedural characteristics** | |  |  |  |  |  |
|  | Index event of PCI | -14.32 | 16.51 | -0.06 | -46.85;18.19 | 0.39 |
| **Time to AA-induced agg** | | 19.18 | 13.89 | 0.09 | 8.17;46.54 | 0.17 |
| Abbreviations: MI, Myocardial infarction; LV-function, Left ventricular-function; TIA, Transient ischemic attack; GI Bleeding, Gastrointestinal Bleeding; PAD, Peripheral artery disease; PCI, Percutaneous coronary intervention | | | | | | |

**Supplemental Table S1. Multiple regression analysis of factors associated with AA-induced aggregation**
